# Developing Universal Genetic Tools for Rapid and Efficient Deletion Mutation in *Vibrio* Species Based on Suicide T-Vectors Carrying a Novel Counterselectable Marker, *vmi480*

**DOI:** 10.1371/journal.pone.0144465

**Published:** 2015-12-07

**Authors:** Peng Luo, Xiangyan He, Qiuting Liu, Chaoqun Hu

**Affiliations:** 1 Key Laboratory of Tropical Marine Bio-resources and Ecology, South China Sea Institute of Oceanology, Chinese Academy of Sciences, Guangzhou, China; 2 Guangdong Key Laboratory of Applied Marine Biology, Chinese Academy of Sciences, Guangzhou, China; 3 South China Sea Bio-Resource Exploitation and Utilization Collaborative Innovation Center, Guangzhou, China; 4 University of Chinese Academy of Sciences, Beijing, China; Imperial College London, UNITED KINGDOM

## Abstract

Despite that *Vibrio* spp. have a significant impact on the health of humans and aquatic animals, the molecular basis of their pathogenesis is little known, mainly due to the limited genetic tools for the functional research of genes in *Vibrio*. In some cases, deletion of target DNAs in *Vibrio* can be achieved through the use of suicide vectors. However, these strategies are time-consuming and lack universality, and the widely used counterselectable gene *sacB* does not work well in *Vibrio* cells. In this study, we developed universal genetic tools for rapid and efficient deletion mutations in *Vibrio* species based on suicide T-Vectors carrying a novel counterselectable marker, *vmi480*. We explored two uncharacterized genes, *vmi480* and *vmi470*, in a genomic island from *Vibrio mimicus* VM573 and confirmed that *vmi480* and *vmi470* constitute a two-component toxin-antitoxin system through deletion and expression of *vmi480* and *vmi470*. The product of *vmi480* exhibited strong toxicity to *Escherichia coli* cells. Based on *vmi480* and the P_BAD_ or P_TAC_ promoter system, we constructed two suicide T-vectors, pLP11 and pLP12, and each of these vectors contained a multiple cloning region with two *Ahd*I sites. Both vectors linearized by *Ahd*I digestion could be stored and directly ligated with purified PCR products without a digestion step. By using pLP11 and pLP12 coupled with a highly efficient conjugation system provided by *E*. *coli* β2163, six genes from four representative *Vibrio* species were easily deleted. By using the counterselective marker *vmi480*, we obtained 3–12 positive colonies (deletion mutants) among no more than 20 colonies randomly selected on counterselection plates. The strategy does not require the digestion of PCR products and suicide vectors every time, and it avoids large-scale screening colonies on counterselective plates. These results demonstrate that we successfully developed universal genetic tools for rapid and efficient gene deletion in *Vibrio* species.

## Introduction

*Vibrio* comprises at least 89 species with validly published names [1]. It is one of the most common bacterial groups in marine environments [1,2]. Although *Vibrio* spp. are of great importance for the remineralization of organic matter in the sea [3], the foremost attention brought to this genus is related to its many pathogenic strains. Until now, at least 12 *Vibrio* species have been reported to be pathogenic to human beings [4], among which *V*. *cholerae*, *V*. *parahaemolyticus* and *V*. *vulnificus* are the most notorious due to their significant threat to human health and seafood safety [4–6]. Some members of *Vibrio* are also causative pathogens for aquatic animals, which often cause enormous economic loss [7–9]. In contrast, some species such as *V*. *alginolyticus* have even been reported as probiotics for shrimp aquaculture [10,11].

Despite that *Vibrio* spp. has a significant impact on the health of humankind and aquatic animals, the molecular basis of their pathogenesis is little known, mainly due to the limited genetic tools available for the functional research of genes in most pathogenic *Vibrio* species [12]. Generally, gene deletion coupled with its complementation is one direct way to assess the function of a gene of interest. One of the most widely used methods is allelic exchange between a target gene and a mutation fragment carried by a suicide vector (plasmid) harboring an antibiotic-resistant cassette and a counterselectable gene [13]. In this way, a suicide vector is driven into recipient cells through conjugation. Under selective stress from an antibiotic environment, recipient cells and the plasmid cannot survive unless the plasmid integrates into a specific site through homologous recombination [14]. As allelic exchange mutants may represent only a small fraction of the transformants and may be difficult to isolate, a counterselectable marker is often instrumental for the acquisition of a deletion mutant [13,14]. In the presence of the counterselective compound, a counterselectable gene promotes the death of the microorganisms harboring this gene, and only the deletion mutant losing the integrated form of the vector through a second homologous recombination and the resultant wild-type clones can survive [14,15].

The *Bacillus subtilis* levansucrase gene *sacB* is now certainly the most commonly used of the different counterselectable markers due to its general efficiency in Gram-negative bacteria and for the simplicity of the counterselection protocol [12,16]. *sacB*-based suicide vectors have been occasionally used for allelic exchange in several *Vibrio* species, such as *V*. *cholerae* [16,17], *V*. *anguillarum* [18], and *V*. *alginolyticus* [19]. However, the use of this gene for allelic exchange in more *Vibrio* species or in other common bacterial species is seriously impeded by the necessary absence of NaCl in the counterselection medium [12]. Milton et al. reported that although they obtained a null *flaA* mutant of *V*. *anguillarum* through the use of a *sacB*-based suicide vector, pDM4, *sacB* did not work well, as both colonies lacking the *flaA* gene and colonies maintaining the integrated vector occurred on sucrose-containing plates [18]. In another case of a deletion mutation in *V*. *vulnificus* by using a *sacB*-based suicide vector, counterselection of sucrose sensitivity was not very efficient, as it was time-consuming and involved the screening of large numbers of colonies to find truly sucrose-resistant colonies [20]. In our laboratory, *sacB-*based pDM4 was also used for a deletion mutation in *V*. *alginolyticus* [19], but it is troublesome to arduously screen deletion mutants from numerous background false-positive colonies (internal communication). These cases strongly support the idea that *sacB* is not an ideal counterselectable marker for allelic exchange in halophilic *Vibrio*. Therefore, it is necessary to explore new counterselectable markers for genetic manipulation in *Vibrio*.

In 2005, Demarre et al. developed a series of pSW suicide plasmids and their cognate *E*. *coli* host strains. These plasmids have small sizes (without *mob* genes) and lack identity with any bacterial chromosome gene [21], which apparently enhances the transfer efficiency of the plasmids and largely reduces incorrect integration. Δ*dapA* and Δ*thyA E*. *coli* strains facilitate the counterselection if they are used in plasmid transfer experiments into markerless recipients [21]. Demarre et al. also verified the high conjugation–recombination frequency when this recombineering system was applied to allelic replacement of *V*. *cholerae* [21]. However, all of these plasmids lack the second necessary counterselectable markers, which limit their further application in complete gene knockout in *Vibrio*.

In the present study, we discovered that two uncharacterized genes, *vmi480* (VMD_06480) and *vmi470* (VMD_06470), in a *V*. *mimicus* strain VM573 (NZ_ACYV01000005) formed a new toxin-antitoxin (TA) system. The toxin gene *vmi480* was introduced into a suicide plasmid constructed from pSW23T and pSW25T-*ccdB*. Finally, we obtained two novel suicide T-Vectors, pLP11 and pLP12, carrying the *vmi480* gene as a counterselectable marker. Through the use of pLP11 and pLP12 coupled with a high-efficiency conjugation system provided by host strain *E*. *coli* β2163, we easily deleted six genes in *V*. *alginolyticus*, *V*. *cholerae*, *V*. *parahaemolyticus*, and *V*. *vulnificus*. Therefore, our results demonstrated that we successfully developed universal genetic tools for a rapid and efficient deletion mutation in *Vibrio* species.

## Results

### Functional Prediction of *vmi480* and *vmi470*

**Fig 1:**
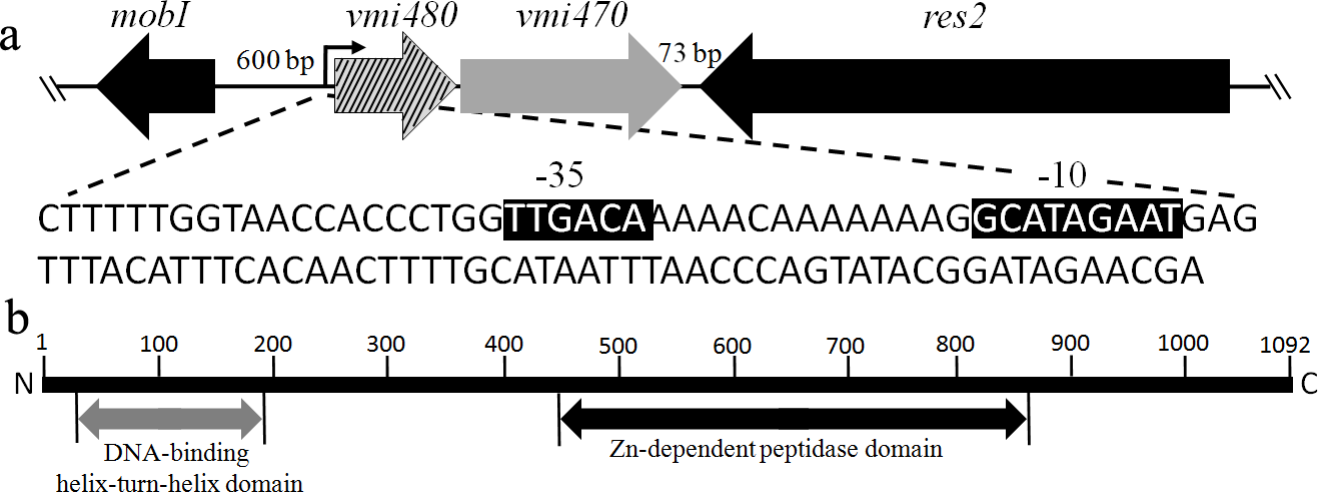
Features of *vmi480* and *vmi470*. **(a)** Schematic diagram of a potential toxin-antitoxin system comprising *vmi480* and *vmi470*. *vmi480* and *vmi470* are located on the same strand and are separated from flanking genes, *mobI* and *res2*. There is a 5-bp intergenic region between *vmi480* and *vmi470*. Upstream of *vmi480*, there is a promoter region for *vmi480* and *vmi470*. These features suggest that *vmi480* and *vmi470* belong to one transcriptional unit and that they are functionally related. The bending arrow represents the position of the promoter, and the white letters on the black background represent the -10 and-35 regions of the promoter. **(b)** Conserved domain analysis of Vmi470. At the N-terminal of Vmi470, there is a DNA-binding helix-turn-helix domain, and at the C-terminal of Vmi470, there is a Zn-dependent peptidase domain.

Previously, we obtained an *E*. *coli* strain LP79 harboring a genomic island, MGI*Vmi*1, from *V*. *mimicus* VM573 through conjugation [22]. MGI*Vmi*1 includes two adjacent genes, *vmi480* and *vmi470*, on the same strand. There is a 5-bp short intergenic region separating two genes, while the genes flanking *vmi480* and *vmi470* are located on the opposite strand and maintain 600-bp and 73-bp intervals from the *vmi480*-*vmi470* locus. There is an intact promoter region upstream of *vmi480* (Fig 1A). These features indicated that *vmi480* and *vmi470* belonged to the same transcriptional unit and were likely functionally related. Blastx searches revealed that *vmi470* encoded a DNA-binding protein; however, conserved domain analysis showed that the product of *vmi470* had a Zn-dependent peptidase domain at the C-terminal region besides a DNA-binding helix-turn-helix domain at the N-terminal region (Fig 1B). Blastx searches also revealed that *vmi480* coded for a hypothetical protein with unknown function and that genes similar to them could only be found in *V*. *mimicus* SX-4, *V*. *anguillarum* 775, a strain from *V*. *cholerae* and a strain from *V*. *ordalii*. In spite of the lack of similarity between two genes and reported toxin-antitoxin genes, the genetic configuration in which *vmi480* and *vmi470* are tightly linked in an operon resemble the findings observed for most type II toxin-antitoxin (TA) systems [23,24]. Then web-based TA prediction was carried on *vmi480* and *vmi470*. The result showed that *vmi480* failed to match any identified toxin genes in database through conserved domain analysis; however the product of *vmi470* contains a DNA-binding helix-turn-helix domain shared by several antitoxins.

### *vmi480* and *vmi470* Constitute a Toxin-Antitoxin Module

**Fig 2:**
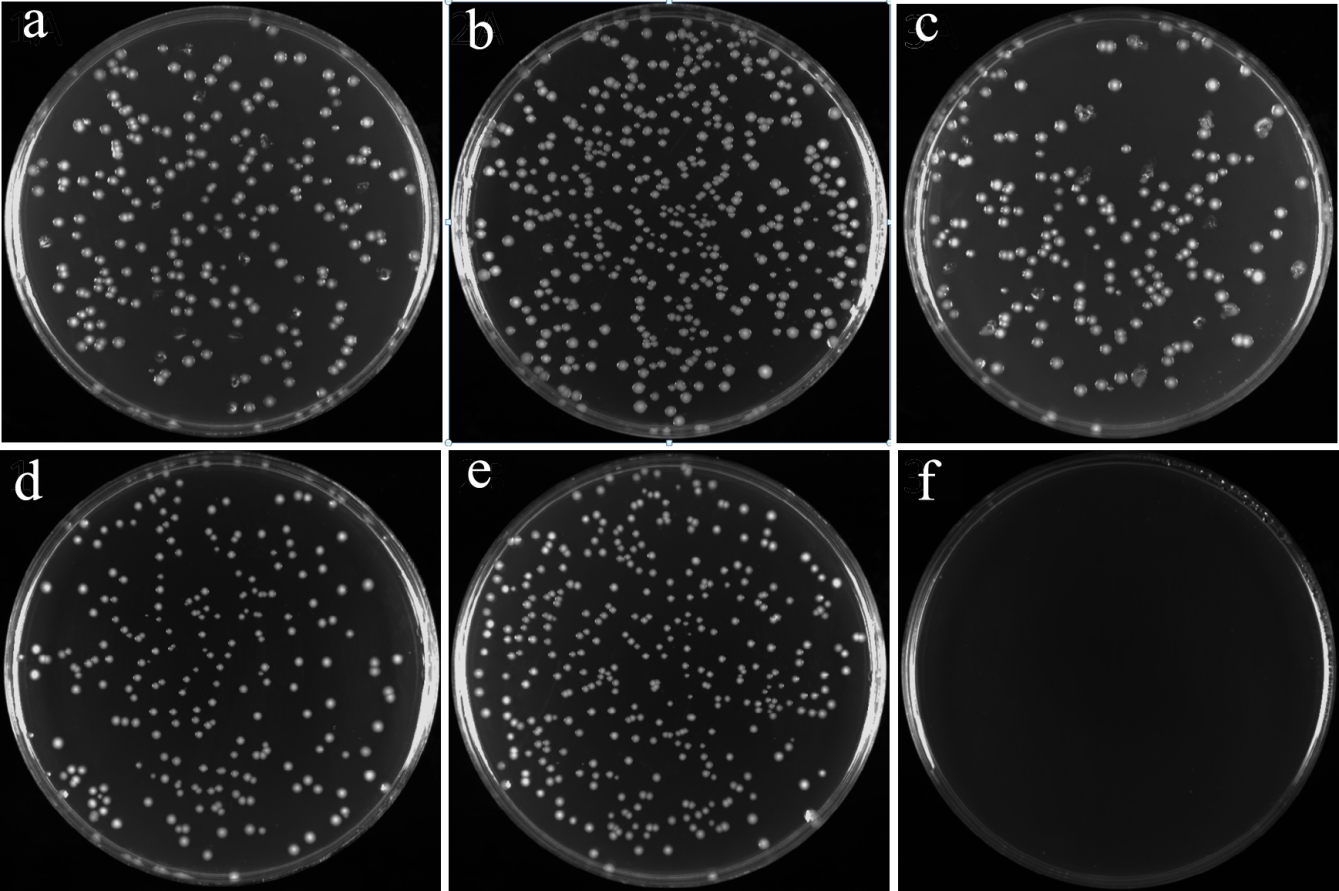
The effect of the expression of *vmi480* and *vmi470* on the growth of *E*. *coli* cells. a, b, c: expression of *vmi480*-*470* (LP134), *vmi470* (LP135), and *vmi480* (LP192) were blocked by D-glucose (0.3%), respectively. d, e, f: expression of *vmi480*-*470* (LP134), *vmi470* (LP135), and *vmi480* (LP192) were activated by L-arabinose (0.2%), respectively.

To further explore the functions of *vmi480* and *vmi470*, we attempted to delete them in *E*. *coli* through one-step inactivation based on the λ recombination system [25]. We easily deleted *vmi480* or both genes but we failed to solely delete *vmi470* in three attempts, which suggest that retaining *vmi480* without *vmi470* may be poisonous to the cells. It raised a concern that *vmi480* and *vmi470* may form a TA pair. *vmi470*, *vmi480* or both genes were further cloned into the expression vector pBAD30. Transformants of *E*. *coli* NEB 5α hosting pBAD30-*vmi480*-*470* (LP134) and pBAD30-*vmi470* (LP135) were easily constructed. In the case of the strain LP134, co-expression of *vmi480* and *vmi470* under the control of P_BAD_ promoter can be achieved as they locate on the same transcription unit. However, a transformant of *E*. *coli* NEB 5α hosting pBAD30-*vmi480* could not be constructed in the initial attempts. This transformant (LP192) was finally constructed by using Luria-Bertani (LB) plates supplemented with D-glucose that blocked the basic expression of *vmi480*. The results showed that basic expression of *vmi480* probably caused some stress on recovered cells, which prevented the formation of the transformant. When blocked by D-glucose or induced by L-arabinose, LP134 (pBAD30-*vmi480*-*470*) and LP135 (pBAD30-*vmi470*) grew well, and no differences were observed between block and induction treatment (Fig 2). When blocked by D-glucose, LP192 (pBAD30-*vmi480*) grew well, but when inactivated by L-arabinose, LP192 did not grow, as no clones were discovered (Fig 2).These results indicated that sole expression of *vmi480* is lethal to the cells but the lethal effect can be eliminated when *vmi480* and *vmi470* are co-expressed in the same vector. It demonstrated that Vmi480 serves as a type of toxin that has a strong lethal effect, while Vmi470 is the antidote of Vmi480. Coupled with the above genetic analysis, the success of deleting *vmi480* or *vmi480*-*vmi470*, and the failure of deleting *vmi470*, these results demonstrate that *vmi480* and *vmi470* compose a two component TA module. An outline of these evidences was shown in Table 1. The strong lethality of *vmi480* product establishes a foundation for applying it as a counterselective marker.

**Table 1:** An outline of evidences that *vmi480* and *vmi470* constitute a TA module.

Aspects	Evidences
**Genetic analysis**	1. *vmi480* and *vmi470* constitute an operon with their own promoter and their genetic configuration resemble those of most type II TA systems.
	2. *vmi470* contains a DNA-binding helix-turn-helix domain shared by several antitoxins.
**Deletion mutants**	3. Deletion of *vmi480* or *vmi480*-*vmi470* can be achieved while deletion of sole *vmi470* cannot fulfill.
**Ectopic expression**	4. Sole expression of *vmi480* is lethal to the cells.
	5. Sole expression of *vmi470* has no effect on cell growth.
	6. Lethality of Vmi480 can be eliminated when both genes are co-expressed in one vector.

### Suicide T-Vectors pLP11-T and pLP12-T for Gene Disruption in *Vibrio*

**Fig 3:**
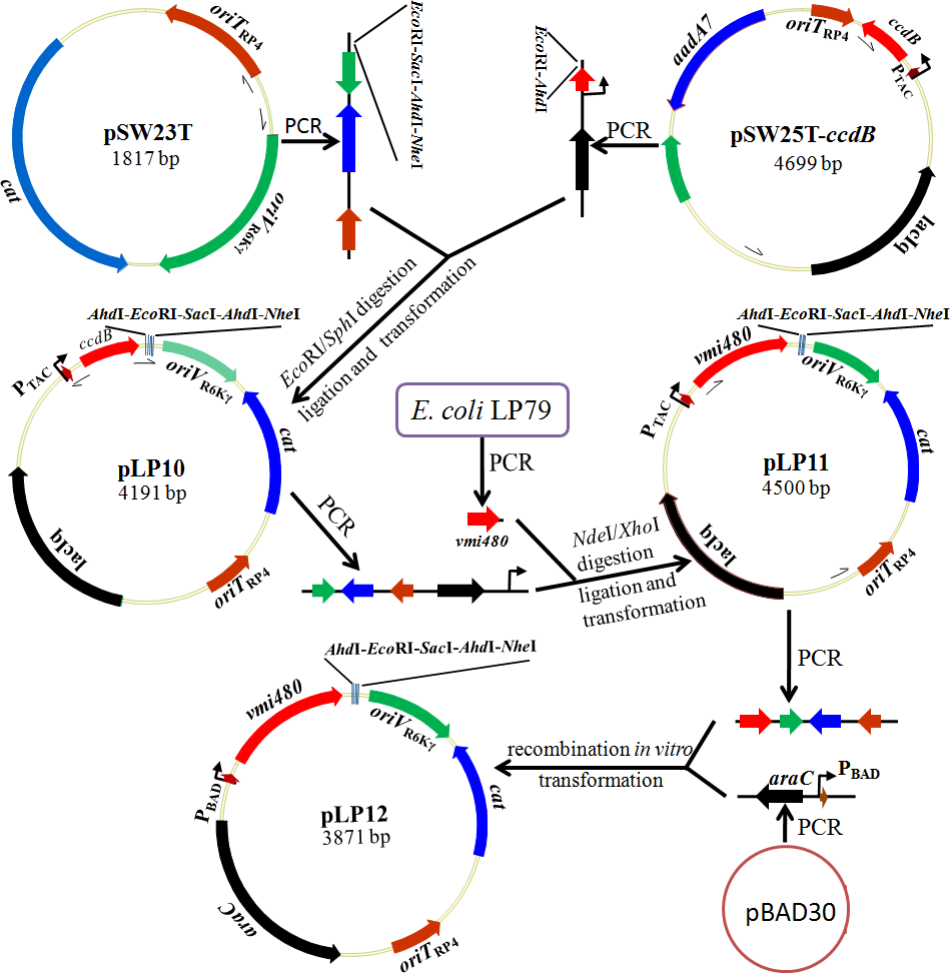
Schematic diagram of the construction of suicide plasmids pLP11 and pLP12.

As shown in Fig 3, we first constructed a derivative plasmid pLP10. pLP10 consists of a fragment containing an R6K origin of replication (*oriV*_R6Kγ_), an RP4 origin of transfer (*oriT*_RP4_) and a chloramphenicol-resistant gene (*cat*) from pSW23T, and a fragment containing a lethal counterselectable gene *ccdB* and a P_TAC_ promoter system from pSW25T-*ccdB*. A multiple cloning site (MCS, *Ahd*I-*Eco*RI-*Sac*I-*Ahd*I-*Nhe*I) was introduced into pLP10 by primers pSW23T-F and pSW25T-F. The *ccdB* gene in pLP10 was replaced by *vmi480* to generate a plasmid pLP11, and then, the P_TAC_ promoter system in pLP11 was replaced by the P_BAD_ promoter system to generate a new plasmid, pLP12. Therefore, the only difference between pLP11 and pLP12 is that the expression of *vmi480* is controlled by the P_TAC_ promoter (pLP11) or by the P_BAD_ promoter (pLP12). Suicide T-vectors, pLP11-T and pLP12-T, were obtained by the amplification of pLP11 and pLP12 following *Ahd*I digestion, which led to each T-vector having single base 3'-T overhangs on both ends. Thus, the overlap of the PCR products of target genes can be purified and directly ligated into linearized pLP11-T or pLP12-T through common TA cloning without digestion steps of overlap PCR products.

### In-Frame Deletion of *hem* in *V*. *alginolyticus*

**Fig 4:**
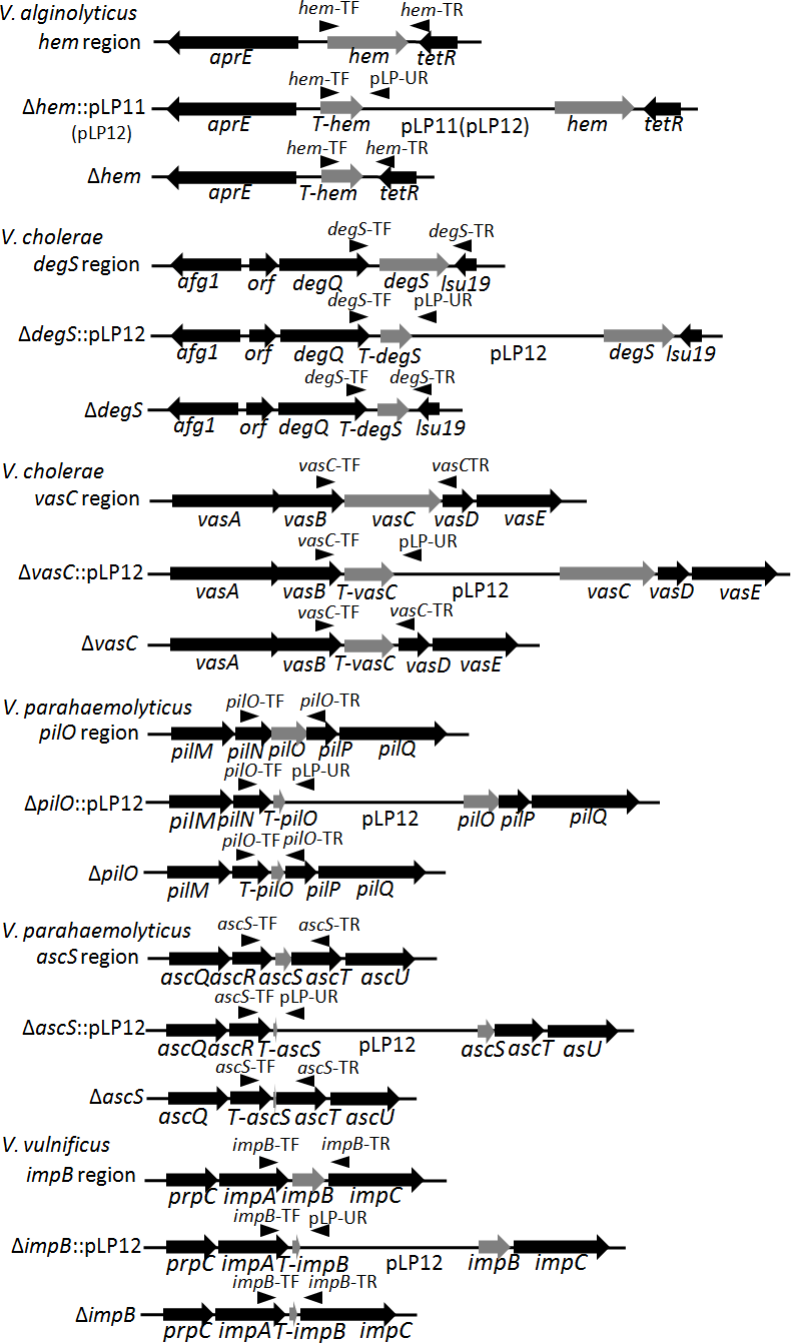
Schematic diagram of wild type, insertional mutation and deletion mutation of targeted genes. Targeted genes are shown with light gray arrows and their adjacent genes are shown with black arrows. Black triangles represent annealing sites of external or internal primers. The gene names prefixed with “T-” represent the names of the truncated genes.

**Fig 5:**
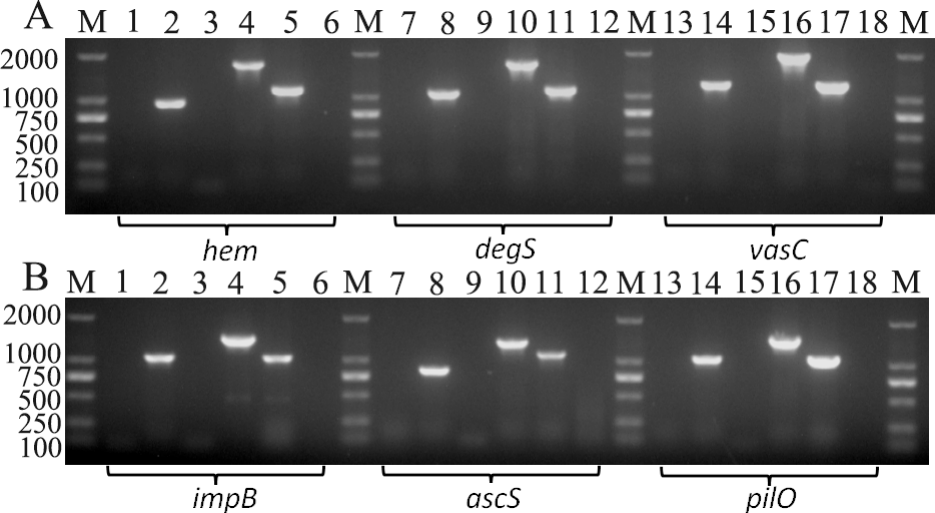
PCR confirmation of insertional disruption and deletion of six genes in four *Vibrio* species. (A) Lane M: DNA marker DL2000; lanes 1–3: PCRs using primers *hem*-TF/pLP-UR to test wild type, insertional disruption and negative control (water as a template) of *hem* in *V*. *alginolyticus* E0601; lanes 4–6: PCRs using primers *hem*-TF/*hem*-TR to test wild type, deletion mutant and negative control (water as a template) of *hem* in *V*. *alginolyticus* E0601 (PCR tests for insertional disruption and deletion of *hem* using pLP11 and pLP12 had the same results, so only the results from pLP11 were shown); lanes 7–9: PCRs using primers *degS*-TF/pLP-UR to test wild type, insertional disruption and negative control (water as a template) of *degS* in *V*. *cholerae* HN375; lanes 10–12: PCRs using primers *degS*-TF/*degS*-TR to test wild type, deletion mutant and negative control (water as a template) of *degS* in *V*. *cholerae* HN375; lanes 13–15: PCRs using primers *vasC*-TF/pLP-UR to test wild type, insertional disruption and negative control (water as a template) of *vasC* in *V*. *cholerae* HN375; lanes 16–18: PCRs using primers *vasC*-TF/*vasC*-TR to test wild type, deletion mutant and negative control (water as a template) of *vasC* in *V*. *cholerae* HN375. (B) Lane M: DNA marker DL2000; lanes 1–3: PCRs using primers *impB*-TF/pLP-UR to test wild type, insertional disruption and negative control (water as a template) of *impB* in *V*. *vulnificus* ATCC 27562; lanes 4–6: PCRs using primers *impB*-TF/*impB*-TR to test wild type, deletion mutant and negative control (water as a template) of *impB* in *V*. *vulnificus* ATCC 27562; lanes 7–9: PCRs using primers *ascS*-TF/pLP-UR to test wild type, insertional disruption and negative control (water as a template) of *ascS* in *V*. *parahaemolyticus* E06135; lanes 10–12: PCRs using primers *ascS*-TF/*ascS*-TR to test wild type, deletion mutant and negative control (water as a template) of *ascS* in *V*. *parahaemolyticus* E06135; lanes 13–15: PCRs using primers *pilO*-TF/pLP-UR to test wild type, insertional disruption and negative control (water as a template) of *pilO* in *V*. *parahaemolyticus* E0680; lanes 16–18: PCRs using primers *pilO*-TF/*pilO*-TR to test wild type, deletion mutant and negative control (water as a template) of *pilO* in *V*. *parahaemolyticus* E0680.

To test the feasibility of our strategy for gene knockout in *Vibrio* species, the *hem* gene (coding hemolysin) from *V*. *alginolyticus* E0601 was first selected as a target gene (Fig 4). Both suicide T-vectors pLP11-T and pLP12-T were used for *hem* knockout. The result showed that both pLP11-*hem* and pLP12-*hem* could integrate into a chromosomal site to form insertional mutants as PCR tests using an external primer *hem*-TF (anchoring upstream of integration site) and an internal primer pLP-UR (anchoring one vector-specific region) revealed that insertional mutants generated one predicted 946-bp band while wild-type E0601 could not as pLP-UR failed match any sites of genomic DNAs from E0601 (only the results from the pLP11-based knockout were shown in Fig 5, as both plasmids aimed at the same gene and led to an identical result). After counterselection driven by toxicity of Vmi480, both suicide vectors could lead to the generation of deletion mutants, as deletion mutants gave rise to one truncated PCR band (1141 bp) while wild-type E0601 gave rise to a normal PCR band (1699 bp) when using two external primers respectively targeting upstream and downstream of *hem* gene (Fig 4). The sequencing of PCR products also confirmed that insertion and deletion mutants were successfully obtained. In the counterselection step, 16 colonies resulting from strain LP204 (integrated pLP11-*hem*) and strain LP206 (integrated pLP12-*hem*) were randomly selected for a PCR test; 3 and 12 colonies were confirmed to be the deletion mutants of the *hem* gene. A higher occurrence of deletion mutants was observed when using the pLP12 plasmid.

### In-Frame Deletion of *degS* and *vasC* in *V*. *cholerae*

To test the feasibility of the genetic tool for gene knockout in *V*. *cholerae*, pLP12-T was used for deletion mutation of *degS* (coding periplasmic serine peptidase) and *vasC* (coding uncharacterized protein in type VI secretion system) in *V*. *cholerae* HN375 (Fig 4). As shown in Fig 5, pLP12-*degS* was integrated into the chromosome, as it led to a 1047-bp PCR fragment when using an external primer *degS*-TF (anchoring upstream of integration site) and the internal vector-specific primer pLP-UR. However, wild-type HN375 could not produce any band when using the same primer pair due to the lack of annealing site by the primer pLP-UR. Under counterselection pressure, the integrated plasmid was removed through a second homologous recombination to form the deletion mutant, which produced a short 1126-bp PCR fragment in the deletion mutant while the wild-type strain HN375 only resulted in a normal band with a size of 1696 bp when using two external primers *degS*-TF/*degS*-TR. As expected, the insertional mutant of *vasC* generated by pLP12-*vasC* led to a 1224-bp PCR fragment but the wild-type strain HN375 could not. The deletion mutant of *vasC* led to a short 1226-bp PCR product while wild-type strain HN375 only generated a normal 1931-bp band. Insertional and deletion mutants of *degS* and *vasC* were also confirmed by subsequent sequencing of PCR products. In the counterselection step, 16 colonies resulting from integrated pLP12-*degS* or pLP12-*vasC* were randomly selected for a PCR test; 8 and 7 colonies were confirmed to be the deletion mutants for *degS* and *vasC*, respectively.

### In-Frame Deletion of *pilO* and *ascS* in *V*. *parahaemolyticus*

pLP12-T was also used for deletion mutation of *pilO* (coding type IV pilus biogenesis protein) and *ascS* (coding preprotein translocase S) in *V*. *parahaemolyticus* (Fig 4). To verify that the method can be used in different strains of the same *Vibrio* species, we aimed to delete *pilO* in *V*. *parahaemolyticus* E0680 and *ascS* in *V*. *parahaemolyticus* E06135. As shown in Fig 5, the result revealed that pLP12-*pilO* integrated into the chromosomal site of E0680 to generate a 1062-bp PCR fragment as expected, while the wild-type strain didn’t result in an amplicon when using primers *pilO*-TF and pLP-UR. After counterselection, the integrated plasmid was removed through a second homologous recombination, and it generated a short 835-bp PCR product representing in-frame deletion. Compared with deletion mutants, wild-type E0680 gave rise to a normal PCR product of 1491 bp when using the same primer pair. Knockout of *ascS* in E06135 had the same outcome. An insertional mutant of *ascS* resulted in one 835-bp PCR fragment and an deletion mutant of the gene resulted in truncated PCR fragment of 1108 bp compared with a normal PCR fragment of 1357 bp from the wild-type strain. PCR results were confirmed by the following sequencing. In the counterselection step, 16 colonies resulting from LP246 (integrated pLP12-*pilO*) or LP248 (integrated pLP12-*ascS*) were randomly selected for a PCR test; 7 and 3 colonies were confirmed to be the deletion mutants of *pilO* and *ascS*, respectively.

### In-Frame Deletion of *impB* in *V*. *vulnificus*

To test the feasibility of the genetic tool for gene knockout in *V*. *vulnificus*, *impB* (coding uncharacterized protein in type VI secretion system) in *V*. *vulnificus* ATCC 27562 was selected as target gene (Fig 4). As shown in Fig 5, pLP12-*impB* integrated into the desired chromosomal site to generate a 1061-bp PCR fragment, while the wild-type strain didn’t. After counterselection, PCR for the clone representing the deletion mutant generated a short 1045-bp DNA fragment as expected, while the wild-type strain generated a normal 1426-bp DNA fragment when using the same external primer pair. The PCR results were confirmed by the following sequencing. In the counterselection step, 20 colonies were randomly selected for a PCR test; 6 colonies resulting from integrated pLP12-*impB* were confirmed to be the deletion mutants. Thus, the results demonstrated that deletion of *impB* was successfully achieved.

## Discussion

Though there are several successful cases of using *sacB*-based suicide vectors for gene knockout in *Vibrio* [e.g., 16–19], SacB toxicity is susceptible to the presence of NaCl in the selective medium [26], while the addition of NaCl to the media is absolutely necessary for the growth of nearly all *Vibrio* species [27].The inadequate toxicity of SacB must largely decrease the occurrence ratio of the correct deletion mutants on counterselective plates, which inevitably increases the difficulty of screening. Therefore, the lack of a suitable counterselective marker has actually become a primary obstacle to genetic manipulation in *Vibrio*. In 2007, Roux et al. constructed a deletion mutant of the *vsm* gene in *V*. *splendidus* by using a *ccdB*-based suicide vector [12]; that study shed some light on the application of a new counterselective marker in the genetic manipulation of *Vibrio* species. However, there is still concern about whether CcdB originated from the F plasmid of *E*. *coli*, which has extensive and strong toxicity in many other *Vibrio* species because CcdB poisons GyrA (the subunit of DNA gyrase) by forming a binding complex [28] while GyrA proteins from *Vibrio* are only distantly related with those from *E*. *coli* strains (comparison by Blastp). In the discrepant intracelluar environment of *Vibrio* (halophilic bacteria), the affinity of CcdB to GyrA in *Vibrio* is likely not as strong as it is in *E*. *coli*. In light of these facts and considerations, exploring new alternative marker genes from rare *Vibrio* strains could help develop genetic tools for gene knockout in *Vibrio*.

First, we aimed to explore the function of *vmi480* and *vmi470* because a Blastx search suggested that *vmi470* codes a DNA-binding protein and its orthologous proteins includes several transcription factors. Thus, we doubted whether *vmi470* plays an important role in the genomic island, MGI*Vmi*1. In another aspect, *vmi480* and *vmi470* constitute an operon with their own promoter and their genetic configuration resemble those of most type II TA systems [23, 24], and thus it also raised a question whether they represent a TA module. In most cases for identification of new TA pairs, gene deletion mutation *in vivo* and ectopic expression of TA genes are key procedures besides theoretical prediction [24,29]. In our case, the attempt to construct a null mutant of a single *vmi470* failed, while deletion of *vmi480* and deletion of *vmi470* and adjacent *vmi480* together were easily achieved. The features of the conserved domains of *vmi470* are similar to antitoxins CcdA and ParD because both of them have a DNA-binding domain at their N-terminal region and have a catalytic domain at their C-terminal region that reacts with their toxins, CcdB and ParE, respectively [30–32]. This suggests that *vmi480* and *vmi470* probably serve as a TA system. Sole expression of *vmi480* was lethal to the cells but the lethal effect could be eliminated when *vmi480* and *vmi470* were co-expressed in the same vector. It clearly showed that the product of *vmi470* serves as an antidote of Vmi480. Generally, co-expression of toxin and antitoxin gene is achieved through adopting two compatible vectors and using different inducers [33–35]. But in our case, we take the full advantages of the genetic structure that not only *vmi480* and *vmi470* are tightly linked (only a 5-bp intergenic region) but also candidate toxin gene *vmi480* locates upstream of candidate antitoxin *vmi470*. It ensures that both genes can be expressed in one expression vector. Suppose that the downstream *vmi470* in the same transcription unit cannot be expressed, Vmi480 will kill cells without the participation of Vmi470. Compared with most of type IITA system, *vmi480*-*vmi470* exhibits two distinct characteristics. One is that candidate toxin consisting of 198 amino acids is encoded by the first gene; this organization is opposite to the widespread TA gene order but occurs in several TA systems such as *higBA* [29,35], *hicAB* [36] and *mqsR*-*ygiT* [24]. Another is that *vmi470* code a candidate antitoxin with a big size of 362 amino acids. Generally, toxin and antitoxin have the small sizes (31–204 amino acids for antitoxins and 41–206 amino acids for toxins) [23]. However, *vmi470* is not the only exception: gene VC0815 from a TA system of *V*. *cholerae* even codes a toxin with much bigger size of 453 amino acids [34]. All these results and analysis well support the idea that *vmi480* and *vmi470* compose a new TA system. These findings actually diversify the TA families and expand our knowledge on TA systems.

Vmi480 exhibited a strong lethal effect to *E*. *coli* cells, and it suggests that although this toxin originates from *Vibrio*, it may have toxicity to bacterial cells from extensive sources. In another aspect, *vmi480* and *vmi470* only exist in rare *Vibrio* strains, which guarantee that *vmi480* is suitable as a counterselection marker for gene deletion in *Vibrio* in most cases. These features of *vmi480* and its product Vmi480 suggest that the *vmi480* gene can be used as an ideal counterselection marker, especially in the construction of suicide vectors. Gaining insight into the mechanism of *vmi480* and *vmi470* is currently ongoing.

Consequently, we constructed suicide vectors based on the plasmid pSW23T and the toxin gene *vmi480* and attempted to use them in the deletion mutation in *Vibrio* strains. *V*. *cholerae*, *V*. *parahaemolyticus* and *V*. *vulnificus* were selected because they are the most harmful *Vibrio* pathogens to humans [4]; thus, there is a more urgent need to develop convenient tools for their genetic manipulation. On the other hand, *V*. *alginolyticus* widely distributes in estuary and marine environments [37,38], and this bacterium has garnered increased concern, as some strains have been reported as pathogenetic to aquatic animals and have caused huge economic losses [4,37,39]. Therefore, *V*. *alginolyticus* was also selected as a desired target for attempted gene knockout. We easily obtained six deletion mutants in five strains from these *Vibrio* species by using new suicide plasmids, pLP11 and pLP12, and the highly efficient conjugation system developed by Demarre et al. [21]. In this conjugation system, donor strain *E*. *coli* β2163 not only contains the transfer apparatus to drive suicide plasmids but also cannot grow on LB plates without thymidine and diaminopimelic acid (DAP) [21]. Therefore, it can minimize the sizes of suicide plasmids and does not require selective markers from the recipient cells [21]. By using this conjugation system coupled with pSW27, *V*. *cholerae* suicide transconjugants were obtained with an overall conjugation–recombination frequency of approximately 2× 10^−7^ transconjugants per recipient [21]. In our experiments, we could obtain 30–70 colonies (suicide transconjugants) on each plate (10^0^ dilution) every time in the first selection (data not shown), which was sufficient to perform the following counterselection. Furthermore, in the counterselection step, we could obtain 3–12 positive colonies (deletion mutants) by randomly screening no more than 20 colonies. The high occurrence of deletion mutants is primarily due to the expression of *vmi480* under induction conditions. As a result, the application of *vmi480* actually saves time for the screening of deletion mutants.

In the conventional strategies of gene deletion through suicide vectors, PCR products and suicide vectors are generally digested by the same restriction enzymes and are then purified before ligation. Occasionally, altering the cloning sites must be performed due to the difference in sequences of inserted DNA fragments. Therefore, the operations are somewhat laborious. To overcome these flaws, we introduced double *Ahd*I sites in MCS and digested pLP11 and pLP12 with *Ahd*I to generate suicide T-vectors. This strategy does not require consideration of restriction sites and evades the laborious steps of digestion and the following purification. In this strategy, purified PCR products are directly ligated with linearized suicide T-vectors. Once linearized suicide T-vectors are prepared, they can be stored and used repeatedly. Therefore, this accelerates the process of gene knockout based on suicide vectors. We do not need to consider the insertional orientation of allelic DNA fragments in suicide vectors because no matter how they are inserted, the suicide vectors carrying allelic DNA fragments will integrate into the desired chromosomal sites in the correct direction due to the homologous recombination mechanism, which will not influence the function of suicide vectors. Of course, we still retain MCS sites in two vectors to meet the preference of different users.

The expression of target genes under the tight control of the P_BAD_ promoter from *E*. *coli* is induced by L-arabinose [40]. To date, the P_BAD_ promoter expression system has been used in many Gram-negative bacteria, such as *E*. *coli*, *Salmonella typhimurium* and *Xanthomonas* sp. [41]. P_BAD_-based suicide vectors or expression vectors were also successfully used in some *Vibrio* species [12,42–44], which suggested that the permeability of L-arabinose may not be a problem in *Vibrio* species. However, there are few reports that P_TAC_-based suicide vectors or expression vectors are used in *Vibrio* cells. Furthermore, the low permeability of isopropyl β-D-1-thiogalactopyranoside (IPTG) has been observed in *Corynebacterium glutamicum* strains, which hampers the application of a P_TAC_-based induction system in this bacterium [41]. It is well known that IPTG acts as an analogue of lactose in expression systems based on the *lac* operon; lactose is mainly transported through lactose permease on the cell membrane of *E*. *coli* [45,46]. However, no lactose permease has been discovered in *Vibrio* to date. This suggests that induction from IPTG in *Vibrio* may not be as efficient as induction in *E*. *coli*. In our case, the loss of integrated pLP11 was not observed in *V*. *alginolyticus* when using 1 mM of IPTG (usual dosage for induction) to induce the expression of *vmi480* (data not shown) until we increased the dosage of IPTG to 5 mM. Given these considerations, although we successfully developed two suicide plasmids carrying *vmi480* and different promoter systems, we prefer to use the suicide plasmid pLP12 containing the P_BAD_ promoter activated by L-arabinose.

Finally, we must note that although our genetic tools were designed to be used in gene disruption in *Vibrio*, they likely have the potential to be applied in other Gram-negative bacteria because the toxicity of Vmi480 may be broad-spectrum to various bacteria and donor strain *E*. *coli* β2163 can conjugate with a wide range of Gram-negative bacteria [21].

## Conclusions

In this study, we confirmed that *vmi480* and *vmi470* in a genomic island, MGI*Vmi*1, from *V*. *mimicus* VM573 belong to a type of two-component toxin-antitoxin system through their knockout and expression. *vmi480* is an uncharacterized toxin gene. We constructed two suicide T-vectors featuring the toxin gene *vmi480*, a P_BAD_ or P_TAC_ promoter system, and a MCS region including two *Ahd*I sites. Linearized vectors by *Ahd*I digestion can be stored and directly ligated with purified PCR products without digestion steps. Using two suicide T-vectors coupled with a highly efficient conjugation system from *E*. *coli* β2163, we easily deleted six genes from four representative *Vibrio* species. Through the use of counterselective marker *vmi480*, we could obtain 3–12 positive colonies (deletion mutants) among no more than 20 randomly selected colonies on counterselection plates. These results demonstrated that we successfully developed universal genetic tools for rapid and efficient gene deletion in *Vibrio* species.

## Materials and Methods

### Bacterial Strains, Plasmids, and Culture Conditions

The bacterial strains and plasmids used in this study are listed in Table 2. *E*. *coli* and *Vibrio* strains were cultured in LB medium. DAP were supplemented to a final concentration of 0.3 mM when necessary. Antibiotics were used at the following concentrations: nalidixic acid (Nx), 40 μg/ml; ampicillin (Ap), 100 μg/ml; spectinomycin (Sp), 50 μg/ml; kanamycin (Kn), 50μg/ml; chloramphenicol (Cm), 20 μg/ml for the propagation of suicide plasmids in host strains, and 10 μg/ml for integrated plasmids in *Vibrio* genomes. Induction of gene expression under the control of the P_BAD_ promoter (from pBAD30 or pLP12) was achieved by the addition of 0.2% L-arabinose to the growth media, and the expression was repressed by the addition of 0.3% D-glucose. Induction of *vmi480* expression under control of the P_TAC_ promoter was carried out by the addition of IPTG to the LB plates at a final concentration of 5 mM.

**Table 2:** Strains and plasmids used in this study.

Strain or plasmid	Description	Reference or source
*E*. *coli*		
DH5α λ*pir*	F^−^ φ80 *lacZ* Δ*M15 recA1 end A1 hsdR17 supE44 thi-1 gyrA96 relA1* (*lacZYA*-*argF*) *U169* λ*pir* lysogen	Laboratory collection
NEB5α		NEB
β2163	(F-) RP4-2-Tc::Mu Δ*dapA*::(*erm*-*pir*)	[21]
LP79	MG1655 MGI*Vmi*1 *res2*::pSW23T (Nx^R^, Cm^R^)	[22]
LP86	MG1655 MGI*Vmi*1 *res2*::pSW23T pKD46 (Nx^R^, Cm^R^, Ap^R^)	This study
LP116	MG1655 MGI*Vmi*1 *res2*::pSW23T Δ*vmi480*-*470* (Nx^R^, Cm^R^)	This study
LP128	MG1655 MGI*Vmi*1 *res2*::pSW23T Δ*vmi480* (Nx^R^, Cm^R^)	This study
LP134	NEB5α pBAD30-*vmi480*-*470* (Ap^R^)	This study
LP135	NEB5α pBAD30-*vmi470* (Ap^R^)	This study
LP192	NEB5α pBAD30-*vmi480* (Ap^R^)	This study
LP194	DH5α λpir pLP10 (Cm^R^)	This study
LP196	DH5α λpir pLP11 (Cm^R^)	This study
LP197	DH5α λpir pLP12 (Cm^R^)	This study
*V*. *alginolyticus*		
E0601	Isolated from seawater in Yangjiang, China	This study
LP204	E0601 *hem*::pLP11 (Cm^R^)	This study
LP206	E0601 *hem*::pLP12 (Cm^R^)	This study
LP207	E0601 Δ*hem* (resulted from LP204)	This study
LP208	E0601 Δ*hem* (resulted from LP206)	This study
*V*. *cholerae*		
HN375	Isolated from seawater in Zhanjiang, China	This study
LP228	HN375 *degS*::pLP12 (Cm^R^)	This study
LP233	HN375 Δ*degS*	This study
LP230	HN375 *vasC*::pLP12 (Cm^R^)	This study
LP235	HN375 Δ*vasC*	This study
*V*. *parahaemolyticus*		This study
E0680	Isolated from an oyster in Yangjiang, China	This study
E06135	Isolated from *Litopenaeus vannamei* in Yangjiang, China	This study
LP246	E0680 *pilO*:: pLP12 (Cm^R^)	This study
LP250	E0680 Δ*pilO*	This study
LP248	E06135 *ascS*:: pLP12 (Cm^R^)	This study
LP252	E06135 Δ*ascS*	This study
*V*. *vulnificus*		This study
ATCC 27562		ATCC
LP239	ATCC 27562 *impB*:: pLP12 (Cm^R^)	This study
LP244	ATCC 27562 Δ*impB*	This study
Plasmids		
pSW23T	*oriT*_RP4_ *oriV*_R6K_ (Cm^R^)	[21]
pSW25T-*ccdB*	*oriT*_RP4_ *oriV*_R6K_ *ccdB* (Sp^R^)	Laboratory collection
pBAD30	*ori*_p15A_ *araC* P_BAD_ (Ap^R^)	[40]
pKD4	PCR template for one-step gene inactivation (Kn^R^)	[25]
pKD13	PCR template for one-step gene inactivation (Kn^R^)	[25]
pKD46	λ- recombination plasmid (Ap^R^)	[25]
pCP20	Helper plasmid to delete resistant gene with FRT sites (Ap^R^)	[25]
pLP10	*oriT*_RP4_ *oriV*_R6K_ *ccdB* P_TAC_ promoter (Cm^R^)	This study
pLP11	*oriT*_RP4_ *oriV*_R6K_ *vmi480* P_TAC_ promoter (Cm^R^)	This study
pLP12	*oriT*_RP4_ *oriV*_R6K_ *vmi480* P_BAD_ (Cm^R^)	This study
pLP11-*hem*	pLP11 containing homologous arms of *hem* gene of E0601	This study
pLP12-*hem*	pLP12 containing homologous arms of *hem* gene of E0601	This study
pLP12-*degS*	pLP12 containing homologous arms of *degS* gene of HN375	This study
pLP12-*vasC*	pLP12 containing homologous arms of *vasC* gene of HN375	This study
pLP12-*pilO*	pLP12 containing homologous arms of *pilO* gene of E0680	This study
pLP12-*ascS*	pLP12 containing homologous arms of *ascS* gene of E06135	This study
pLP12-*impB*	pLP12 containing homologous arms of *impB* gene of ATCC 27562	This study

### PCR, Sequencing and Other Molecular Methods

PCR assays were performed using the primers described in S1 Table. When PrimSTAR Max DNA Polymerase (Takara, China) was used, PCR conditions were as follows: 2 min at 98°C; 30 cycles of 10 sec at 98°C, 10 sec at the appropriate annealing temperature, 30 sec/kb at 72°C; and 5 min at 72°C. When rTaq DNA Polymerase (Takara) was used, PCR conditions were as follows: 4 min at 94°C; 30 cycles of 20 sec at 94°C, 30 sec at the appropriate annealing temperature, 1 min/kb at 72°C; and 7 min at 72°C. When necessary, PCR products were purified using a DNA Purification and Concentration Kit (ZhongDing, China). When necessary, PCR products were also sent to the company (BGI, China) for direct sequencing. Plasmids were extracted using the PureYield Plasmid Miniprep System (Promega, USA) according to the manufacturer’s instruction.

### Construction of Null Mutants for *vmi480*, *vmi470* and *vmi480-470* in *E*. *coli*

*E*. *coli* LP79 was first transformed with the recombination plasmid pKD46 by electroporation according to Dower et al. [47]. Electroporation was carried out in a BioRad Gene Pulser Xcell apparatus set at 25 μF and 1.8 kV using 1-mm gap electroporation cuvettes. One-step inactivations of *vmi480*, *vmi470*, and *vmi480*-*470* in the transformant LP86 (carrying pKD46) were carried out with the protocol from Datsenko et al. [25]. pKD4 and pKD13 were used to generate PCR fragments containing homologous arms of the abovementioned genes and a kanamycin-resistant cassette.

### Construction of Expression Vectors of *vmi480*, *vmi470* and *vmi480*-*470*

Complete genes of *vmi480*, *vmi470* and *vmi480*-*470* were amplified by primer pairs, 480-exF/480-exR, 470-exF/470-exR and 480-470-exF/480-470-exR, respectively. PCR products of *vmi480*, *vmi470* and *vmi480*-*470* were purified and digested with *Eco*R I and *Xba* I, and they were ligated with vector pBAD30 digested with the same restriction enzymes. The ligation products were transformed into competent *E*. *coli* NEB5a cells (NEB) according to the manufacturer’s instructions. During the construction of the transformant hosting the plasmid pBAD30-*vmi480*, D-glucose was added into the medium to repress expression of *vmi480*. Transformants were screened on LB plates supplemented with Ap. Transformants were identified by PCR using the primer pair pBAD30-TF/pBAD30-TR and were confirmed by subsequent sequencing.

### Assay of the Lethal Effect of Toxin Vmi480 and Its Antidote Vmi470

To test the lethal effect of Vmi480 and the antagonism of Vmi470 against Vmi480, transformants of *E*. *coli* LP134 (pBAD30-*vmi480*-*470*), LP135 (pBAD30-*vmi470*) and LP192 (pBAD30-*vmi480*) were grown in LB broth supplemented with D-glucose at 37°C for 6 hr and serially diluted. Samples (10^−4^ dilution) were spread on LB plates with D-glucose or with L-arabinose, and they were then incubated at 37 C overnight.

### Construction of Suicide T-Vectors carrying the Lethal Gene *vmi480*

In the process of constructing suicide T-vectors, pSW23T and pSW25T-*ccdB* were used as the initiator plasmids. PCR of pSW23T was carried out using the primer pair pSW23T-F/pSW23T-R to generate a fragment containing *oriV*_R6Kγ_, *oriT*_RP4_ and *cat*. PCR of pSW25T-*ccdB* was carried out using the primer pair pSW25T-F/pSW25T-R to generate a fragment containing *ccdB*, *lacIq* and P_TAC_. A multiple cloning site (*Ahd*I-*Eco*RI-*Sac*I-*Ahd*I-*Nhe*I) was introduced by primers pSW23T-F and pSW25T-F. Two fragments were purified, digested by *Eco*RI and *Sph*I, and ligated together. The ligation product was transformed into *E*. *coli* DH5α λ pir cells to obtain a strain LP194 hosting a resultant plasmid, pLP10. Chemical transformations were performed according to the method by Swords [48]. The construction of pLP10 was tested by PCR with two primer pairs, pLP10L-TF1/ pLP10L-TR1 and pLP10L-TF2/ pLP10L-TR2, and confirmed by sequencing.

A fragment containing all the parts of pLP10 except *ccdB* was acquired by reverse PCR amplification from pLP10 using primers pLP10-F and pLP10-R. *vmi480* was amplified from *E*. *coli* LP79 using primers *vmi480*-F and *vmi480*-R. They were purified, digested by *Nde*I and *Xho*I, and ligated together, and then the ligation product was transformed into *E*. *coli* DH5α λ pir cells to obtain a strain LP196 hosting a resultant plasmid, pLP11. The construction of pLP11 was confirmed by the primer pair pLP11L-TF/pLP11L-TR followed by sequencing.

pLP11 was amplified with primers pLP11-F and pLP11-R to generate a fragment retaining all the parts of the plasmid except the P_TAC_ promoter and *lacIq* gene. The P_BAD_ promoter system was amplified from plasmid pBAD30 using primers pBAD30-PF and pBAD30-PR. Two fragments were recombined together through *in vitro* recombination using ClonExpress® II One Step Cloning Kit (Vazyme, China), where recombinase Exnase was used to avoid the excess introduction of restriction sites into the resulting plasmid. The recombination product was transformed into *E*. *coli* DH5α λ pir cells to obtain a strain LP197 hosting the resultant plasmid, pLP12. The construction of pLP12 was confirmed by PCR testing with the primer pair pLP12L-TF/pLP12L-TR followed by sequencing.

To avoid repetitive extraction of plasmids for generating suicide T-vectors and to decrease the false clones in the subsequent construction of recombinant T-vectors, pLP11 and pLP12 were amplified using PrimSTAR Max DNA Polymerase and the primer pair STVU-F/STVU-R. PCR products were digested by *Ahd*I and purified to finally obtain the linearized suicide T-vectors pLP11-T and pLP12-T.

### Construction of Deletion Mutants of Six Genes from Four *Vibrio* Species

Six genes from four representative *Vibrio* species were targeted for deletion mutation. In-frame deletion fragments consisting of two flanking regions of each target locus were made by overlap extension PCR [19]. PrimSTAR Max DNA Polymerase was adopted in the first PCR, and rTaq DNA polymerase was used in the second PCR to conveniently add the single base A to the 3' end of the PCR products. The final PCR products were purified and ligated with pLP11-T or pLP12-T. The ligation products were transformed into competent *E*. *coli* DH5α λ pir cells to generate recombinant suicide plasmids carrying these homologous fragments for allelic exchange of targeted genes. Recombinant plasmids were extracted and transformed into *E*. *coli* β2163 by electroporation. Then, the recombinant plasmids were transferred into *Vibrio* strains through conjugation.

Conjugations were performed by mixing equal volumes of recombinant *E*. *coli* β2163 and each *Vibrio* strain grown overnight at 37°C. The cells were harvested by centrifugation for 2 min at 8000 g, washed in 400 μL of LB broth and resuspended in 10 μL of LB broth. Mating mixtures were then deposited on LB plates supplemented with DAP and D-glucose and incubated at 37°C for 8 hr. The cells were recovered from the plates in 1 ml of LB broth. Each of the 100-μL of mixed cells was spread on LB plates supplemented with Cm and D-glucose for screening of single-crossover cells with integrated plasmids into specific chromosomal loci. The clones were purified on the same LB plates to make sure that stable and correct insertional mutants were obtained. Then, these insertional mutants were checked by PCR with external primers targeting upstream of integration sites and an internal primer targeting vector-specific region. In this condition, wild-type strains will not result in any predicted PCR bands. Insertional mutants were grown at 37°C for 6 hr, serially diluted, and spread on LB plates supplemented with IPTG or L-arabinose for counterselection of deletion mutants (double-crossover recombination). The clones on counterselection plates were randomly selected and purified before PCR assays. Two external primers respectively anchoring upstream and downstream of targeted genes were adopted for the detection of deletion mutants. All of the insertional mutants and deletion mutants were confirmed by PCR using the same primer pair and subsequent sequencing.

### Bioinformatics Analysis and Nucleotide Sequence Accession Numbers

Blastx searches against *vmi480* and its adjacent *vmi470* were performed to predict their function. The promoter was predicted by online tools (http://molbiol-tools.ca/Promoters.htm). A web-based TA prediction tool was adopted to identify *vmi480* and *vmi470* (http://genoweb1.irisa.fr/duals/RASTA-Bacteria/index.php?page=form). The sequences of the suicide plasmids pLP11 and pLP12 have been deposited at GenBank under accession numbers KT326152 and KT326153, respectively.

## Supporting Information

S1 Table Primers used in this study. (DOCX)

## References

[1] Jin C, Luo P, Zuo H, Chen J, Chen M, Wang W. *Vibrio Zhuhaiensis* sp. nov., isolated from Japanese prawn (*Marsupenaeus japonicus*). Antonie van Leeuwenhoek. 2013; 103:989–96. 10.1007/s10482-013-9878-4 23338602

[2] Howard RJ, Bennett NT. Infections caused by halophilic marine *Vibrio* bacteria. Ann Surg. 1993; 217:525–31. 8489315 10.1097/00000658-199305010-00013 PMC1242837

[3] Fukami K, Simidu U, Taga N. Microbial decomposition of phyto- and zooplankton in seawater. II. Changes in the bacterial community. Mar Ecol Prog Ser. 1985; 21:7–23.

[4] Daniels NA, Shafaie A. A review of pathogenic *Vibrio* infections for clinicians. Infect Med. 2000; 17:665–85.

[5] Bhunia AK. Vibrio cholerae, V. parahaemolyticus, V. vulnificus In: Bhunia AK, editor. Foodborne microbial pathogens. Springer; 2008 p. 241–52.

[6] Turner JW, Malayil L, Guadagnoli D, Cole D, Lipp EK. Detection of *Vibrio parahaemolyticus*, *Vibrio vulnificus* and *Vibrio cholerae* with respect to seasonal fluctuations in temperature and plankton abundance. Environ Microbiol. 2014; 16:1019–28. 10.1111/1462-2920.12246 24024909

[7] Lafferty KD, Harvell CD, Conrad JM, Friedman CS, Kent ML, Kuris AM, et al Infectious diseases affect marine fisheries and aquaculture economics. Ann Rev Mar Sci. 2015; 7:471–96. 10.1146/annurev-marine-010814-015646 25251276

[8] Mahbub KR, Paul KP, Ahmed MM. Prevalence of *Vibrio* spp. and antibiogram of isolates from shrimp rearing ponds in Bangladesh. J Adv Sci Res. 2011; 2:74–80.

[9] Shruti C, Soumya H. *Vibrio* related diseases in aquaculture and development of rapid and accurate methods. J Mar Sci Res Dev. 2012; S1:002.

[10] Vandenberghe J, Thompson FL, Gomez-Gill B, Swings J. Phenotypic diversity amongst *Vibrio* isolates from marine aquaculture systems. Aquaculture. 2003; 219:9–20.

[11] Direkbusaram S, Yoshimizu M, Ezura Y, Ruangpan L, Danayadol Y. *Vibrio* spp., the dominant flora in shrimp hatchery against some fish pathogenic viruses. J Mar Biotech. 1998; l6:266–67. 9852624

[12] Roux FL, Binesse J, Soulnier D, Mazel D. Construction of a *Vibrio splendidus* mutant lacking the metalloprotease gene *vsm* by use of a novel counterselectable suicide vector. Appl Environ Microbiol. 2007; 73:777–84. 17122399 10.1128/AEM.02147-06 PMC1800747

[13] Nakashima N, Miyazaki K. Bacterial cellular engineering by genome editing and gene silencing. Int J Mol Sci. 2014; 15:2773–93. 10.3390/ijms15022773 24552876 PMC3958881

[14] Reyrat J, Pelicic V, Gicquel B, Rappuoli R. Counterselectable markers: untapped tools for bacterial genetics and pathogenesis. Infect Immun. 1998; 66:4011–17. 9712740 10.1128/iai.66.9.4011-4017.1998 PMC108478

[15] Stibitz S. Use of conditionally counterselectable suicide vectors for allelic exchange. Methods Enzymol. 1994; 235:458–65. 8057916 10.1016/0076-6879(94)35161-9

[16] Donnenberg MS, Kaper JB. Construction of an *eae* deletion mutant of enteropathogenic *Escherichia coli* by using a positive-selection suicide vector. Infect Immun. 1991; 59:4310–17. 1937792 10.1128/iai.59.12.4310-4317.1991 PMC259042

[17] Karlsson SL, Ax E, Nygren E, Källgård S, Blomquist M, Ekman A, et al Development of stable *Vibrio cholerae* O1 Hikojima type vaccine strains co-expressing the Inaba and Ogawa lipopolysaccharide antigens. PLoS One. 2014; 9:e108521 10.1371/journal.pone.0108521 25397871 PMC4232259

[18] Milton DL, OToole R, Hörstedt P, Wolf-Watz H. Flagellin A is essential for the virulence of *Vibrio anguillarum* . J Bacteriol. 1996; 178:1310–19. 8631707 10.1128/jb.178.5.1310-1319.1996 PMC177804

[19] Chen C, Zhao JJ, Ren CH, Hu CQ. Deletion of *valR*, a homolog of *Vibrio harveyi luxR* generates an intermediate colony phenotype between opaque/rugose and translucent/smooth in *Vibrio alginolyticus* . Biofouling. 2010; 26:595–601. 10.1080/08927014.2010.499511 20582761

[20] Gulig PA, Tucker MS, Thiaville PC, Joseph JL, Brown RN. USER friendly cloning coupled with chitin-based natural transformation enables rapid mutagenesis of *Vibrio vulnificus* . Appl Environ Microbiol. 2009; 75:4936–49. 10.1128/AEM.02564-08 19502446 PMC2725515

[21] Demarre G, Guérout AM, Matsumoto-Mashimo C, Rowe-Magnus DA, Marlière P, Mazel D. A new family of mobilizable suicide plasmids based on broad host range R388 plasmid (IncW) and RP4 plasmid (IncPα) conjugative machineries and their cognate *Escherichia coli* host strains. Res Microbiol. 2005; 156:245–55. 15748991 10.1016/j.resmic.2004.09.007

[22] Carraro N, Matteau D, Luo P, Rodrigue S, Burrus V. The master activator of IncA/C conjugative plasmids stimulates genomic islands and multidrug resistance dissemination. PLoS Genet. 2014; 10:e1004714 10.1371/journal.pgen.1004714 25340549 PMC4207636

[23] Pandey DP, Gerdes K. Toxin-antitoxin loci are highly abundant in free-living but lost from host-associated prokaryotes. Nucleic Acids Res. 2005; 33:966–76. 15718296 10.1093/nar/gki201 PMC549392

[24] Kasari V, Kurg K, margus T, Tenson T, Kaldalu N. The *Escherichia coli mqsR* and *ygiT* genes encode a new toxin-antitoxin pair. J Bacteriol. 2010; 192:2908–19. 10.1128/JB.01266-09 20233923 PMC2876487

[25] Datsenko KA, Wanner BL. One-step inactivation of chromosomal genes in *Escherichia coli* K-12 using PCR products. Proc Natl Acad Sci USA. 2000; 97: 6640–45. 10829079 10.1073/pnas.120163297 PMC18686

[26] Blomfield IC, Vaughn V, Rest RF, Eisenstein BI. Allelic exchange in *Escherichia coli* using the *Bacillus subtilis sacB* gene and a temperature-sensitive pSC101 replicon. Mol Microbiol. 1991; 5:1447–57. 1686293 10.1111/j.1365-2958.1991.tb00791.x

[27] Baumann P, Schubert RHW. Genus II. Vibrionaceae Veron 1965 In: Krieg NR, Holt JG, editors. Bergey’s manual of systematic bacteriology, vol 1 Baltimore: Williams & Wilkins 1984; p. 516–17.

[28] Kampranis SC, Howells AJ, Maxwell A. The interaction of DNA gyrase with the bacterial toxin CcdB: evidence for the existence of two gyrase-CcdB complexes. J Mol Biol. 1999; 293:733–44. 10543963 10.1006/jmbi.1999.3182

[29] Budde PP, Davisn BM, Yuan J, Waldor MK. Characterization of a *higBA* toxin-antitoxin locus in *Vibrio cholerae* . J Bacteriol. 2007; 189: 491–500. 17085558 10.1128/JB.00909-06 PMC1797405

[30] Bernard P, Couturier M. The 41 carboxy-terminal residues of the mini F plasmid CcdA protein are sufficient to antagonize the killer activity of the CcdB protein. Mol Gen Genet. 1991; 226:297–304. 2034222 10.1007/BF00273616

[31] Madl T, Van Melderen L, Mine N, Respondek M, Oberer M, Keller W, et al Structural basis for nucleic acid and toxin recognition of the bacterial antitoxin CcdA. J Mol Biol. 2006; 364:170–85. 17007877 10.1016/j.jmb.2006.08.082

[32] Oberer M, Zangger K, Gruber K, Keller W. The solution structure of ParD, the antidote of the ParDE toxin–antitoxin module, provides the structural basis for DNA and toxin binding. Protein Sci. 2007; 16:1676–88. 17656583 10.1110/ps.062680707 PMC2203376

[33] Leplae R, Geeraerts D, Hallez R, Guglielmini J, Drèze P. Diversity of bacterial type II toxin-antitoxin systems: a comprehensive search and functional analysis of novel families. Nucleic Acids Res. 2011; 39:5513–25. 10.1093/nar/gkr131 21422074 PMC3141249

[34] Iqbal N, Guérout A, Krin E, Roux FL, Mazel D. Comprehensive functional analysis of the 18 *Vibrio cholerae* N16961 toxin-antitoxin systems substantiates their role in stabilizing the superintegron. J Bacteriol. 2015; 197:2150–59. 10.1128/JB.00108-15 25897030 PMC4455273

[35] Christensen-Dalsgaard M, Gerdes K. Two *higBA* loci in the *Vibrio cholerae* superintegron encode mRNA cleaving enzymes and can stabilize plasmids. Mol Microbiol. 2006; 62:397–411. 17020579 10.1111/j.1365-2958.2006.05385.x

[36] Jorgensen MG, Pandey DP, Jaskolska M, Gerdes K. HicA of *Escherichia coli* defines a novel family of translation-independent mRNA interferases in bacteria and archaea. J Bacteriol. 2009; 191:1191–99. 10.1128/JB.01013-08 19060138 PMC2631989

[37] Luo P, Hu CQ. Analysis of *gyrB* sequences of *Vibrio alginolyticus* and *gyrB*-targeted rapid PCR identification of the bacterium from environmental isolates. Dis Aquat Org. 2008; 82:209–16. 10.3354/dao01984 19244972

[38] Reilly GD, Reilly CA, Smith EG, Baker-Austin C. *Vibrio alginolyticus*-associated wound infection acquired in British waters, Guernsey, July 2011. Euro Surveill. 2011; 16:19994 22027377

[39] Liu CH, Cheng W, Hsu JP, Chen JC. *Vibrio alginolyticus* infection in the white shrimp *Litopenaeus vannamei* confirmed by polymerase chain reaction and 16S rDNA sequencing. Dis Aquat Org. 2004; 61:169–74. 15584425 10.3354/dao061169

[40] Guzman LM, Belin D, Carson MJ, Beckwith J. Tight 342 regulation, modulation, and high-level expression by vectors containing the 343 arabinose P_BAD_ promoter. J Bacteriol. 1995; 177:4121–30. 7608087 10.1128/jb.177.14.4121-4130.1995 PMC177145

[41] Zhang Y, Shang X, Lai SJ, Zhang GQ, Liang Y, Wen TY. Development and application of an arabinose-inducible expression system by facilitating inducer uptake in *Corynebacterium glutamicum* . Appl Environ Microbiol. 2012; 78:5831–38. 10.1128/AEM.01147-12 22685153 PMC3406113

[42] Yamaichi Y, Fogel MA, Waldor MK. Par genes and the pathology of chromosome loss in *Vibrio cholerae* . Proc Natl Acad Sci USA. 2007; 104:630–35. 17197419 10.1073/pnas.0608341104 PMC1760642

[43] Bordeleau E, Brouillette E, Robichaud N, Burrus V. Beyond antibiotic resistance: integrating conjugative elements of the SXT/R391 family that encode novel diguanylate cyclases participate to c-di-GMP signaling in *Vibrio cholerae* . Environ Microbiol. 2010; 12:510–23. 10.1111/j.1462-2920.2009.02094.x 19888998

[44] Lo Scrudato M, Blokesch M. A transcriptional regulator linking quorum sensing and chitin induction to render *Vibrio cholerae* naturally transformable. Nucleic Acids Res. 2013; 41:3644–58. 10.1093/nar/gkt041 23382174 PMC3616704

[45] Hansen LH, Knudsen S, Sørensen SJ. The effect of the *lacY* gene on the induction of IPTG inducible promoters, studied in *Escherichia coli* and *Pseudomonas fluorescens* . Curr Microbiol. 1998; 36:341–47. 9608745 10.1007/s002849900320

[46] Marbach A, Bettenbrock K. Lac operon induction in *Escherichia coli*: systematic comparison of IPTG and TMG induction and influence of the transacetylase LacA. J Biotech. 2012; 1578:82–8. 10.1016/j.jbiotec.2011.10.009 22079752

[47] Dower WJ, Miller JF, Ragsdale CW. High efficiency transformation of *E*. *coli* by high voltage electroporation. Nucleic Acids Res. 1988; 16:6127–45. 3041370 10.1093/nar/16.13.6127 PMC336852

[48] Swords WE. Chemical transformation of *E*. *coli* . Methods Mol Biol. 2003; 235:49–53. 12904644 10.1385/1-59259-409-3:49

