# Supplementary material for: Developing Universal Genetic Tools for Rapid and Efficient Deletion Mutation in Vibrio Species Based on Suicide T-Vectors Carrying a Novel Counterselectable Marker, vmi480
Source: PLoS One. 2015 Dec 7;10(12):e0144465. doi: 10.1371/journal.pone.0144465 (PMC4671572; doi:10.1371/journal.pone.0144465)
Supplement: S1 Table — (DOCX) [file pone.0144465.s001.docx]

Table S1 Primers used in this study

| Primer name | Nucleotide sequence (5′ to 3′) | Purpose of amplification |
| --- | --- | --- |
| 480-470-MF | TTTTGCATAATTTAACCCAGTATACGGATAGAACGAGTGTAGGCTGGAGCTGCTTCG | Kanamycin-resistant gene flanked by homologous arms outside *vmi480*-*470* |
| 480-470-MR | CTTAAGATGGGTTACTAGTTGGCCATTTAAGATAGTATTCCGGGGATCCGTCGACC |  |
| 480-MF | TTTTGCATAATTTAACCCAGTATACGGATAGAACGAGTGTAGGCTGGAGCTGCTTCG | Kanamycin-resistant gene flanked by homologous arms outside *vmi480* |
| 480-MR | CATTTCTTTTACTCATCAGAGACCTTTTTTTGTACTCATATGAATATCCTCCTTA |  |
| 470-MF | GAAAGTACAAAAAAAGGTCTCTGATGAGTAAAAGAAGTGTAGGCTGGAGCTGCTTCG | Kanamycin-resistant gene flanked by homologous arms outside *vmi470* |
| 470-MR | CTTAAGATGGGTTACTAGTTGGCCATTTAAGATAGTATTCCGGGGATCCGTCGACC |  |
| 480-TMF | CGACTTCTCAATGCAGGTTATC | Test insertional and deletion mutant of *vmi480* |
| 480-TMR | AGACCAGCAGCTACTCGCGCCA |  |
| 480-470-TMF | CGACTTCTCAATGCAGGTTATC | Test insertional and deletion mutant of *vmi480*-*470* |
| 480-470-TMR | CGTCGAATAGAATCTGTTGCG |  |
| 480-470-exF | ACGTATGAATTCAGGAGGAATTCACCATGACCAAAAAACCTGAATTTTAT* | Get complete genes of *vmi480* and *vmi470* for cloning |
| 480-470-exR | CAGCTATCTAGATTAAACGGAACGCAAAGGTG |  |
| 480-exF | ACGTATGAATTCAGGAGGAATTCACCATGACCAAAAAACCTGAATTTTAT | Get complete gene of *vmi480* for cloning |
| 480-exR | CAGCTATCTAGATTACTCATCAGAGACCTTTTTTTG |  |
| 470-exF | ACGTATGAATTCAGGAGGAATTCACCATGATTTATCAAGCTGTTAATC | Get complete gene of *vmi470* for cloning |
| 470-exR | CAGCTATCTAGATTAAACGGAACGCAAAGGTG |  |
| pBAD30-TF | CGTCACACTTTGCTATGCCAT | Test the cloned genes in pBAD30 |
| pBAD30-TR | GCAGTTCCCTACTCTCGCAT |  |
| pSW23T-F | CAGTCAGAATTCGAGCTCGACATATCGTCGCTAGCTGTTCCCTTTAGTGAGGGTT | Get a fragment containing *oriV*_R6Kγ_, *oriT*_RP4_, *cat* and multiple restriction sites |
| pSW23T-R | TCAGTCGCATGCCAAGCTTATCGATACCGTCGA |  |
| pSW25T-F | TAGACTGAATTCGACTCAAGGTCTAGATTCCTGCAGCCCGGGGGAT | Get a fragment containing P_TAC_ promoter system, *ccdB* and another *Ahd*I site |
| pSW25T-R | TGGCCAGTGCCAAGCTTGCA |  |
| pLP10L-TF1 | AGCCATCGGAAGCTGTGGT | Test the correct construction of pLP10 |
| pLP10L-TR1 | CGGCGTAGAGGATCTGAAG |  |
| pLP10L-TF2 | TTATTCTGCGAAGTGATCTTCC |  |
| pLP10L-TR2 | ACGGTGAAAACCTCTGACA |  |
| pLP10-F | ATCAGTCTCGAGGGAATATAAGTCGACGGATC | Get a amplicon containing the whole pLP10 except *ccdB* |
| pLP10-R | GTGTCAATAATGTCACTCTG |  |
| vmi480-F | GATATACATATGATGACCAAAAAACCTGAATTTTATG | Get complete *vmi480* gene with restriction sites |
| vmi480-R | AGTACACTCGAGCTTTTACTCATCAGAGACCTT |  |
| pLP11L-TF | GGCTCACACTTGCCAACCAAACA | Test the correct construction of pLP11 |
| pLP11L-TR | TGCGCGCAATTAACCCTCAC |  |
| pLP11-F | AAGAAGGAGATATACATATGATGACC | Get a amplicon containing the whole pLP11 except P_TAC_ system |
| pLP11-R | CTGTAAGCGGATGCCGGGAG |  |
| pBAD30-PF | CTCCCGGCATCCGCTTACAGATCGATGCATAATGTGCCTGTC | Get a amplicons containing P_BAD_ system and two homologous arms facilitating *in vitro* recombination with the amplicon from pLP11 |
| pBAD30-PR | CATATGTATATCTCCTTCTTCCAAAAAAACGGGTATGGAGAAACAG |  |
| pLP12L-TF | TGACGACCGTAGTGATGAATC | Test the correct construction of pLP12 |
| pLP12L-TR | GACCAGTTACAACTGTGTCGT |  |
| STVU-F | AGTCATGACATATCGTCGCTAGCTGTTCC | Obtain amplicons from pLP11 and pLP12 for generating suicide T-vectors after *Ahd*I digestion |
| STVU-R | GACTTACGACTCAAGGTCTAGATTCCTGC |  |
| *hem*-MF1 | ACTGCATTACTTCCGCTTGCC | Get DNA fragment containing two homologous regions of *hem* gene through overlap PCR |
| *hem*-MR1 | CAATACTTTCGCACGAATCGTGTCTGCGTTCTCGTTCGCTA |  |
| *hem*-MF2 | TAGCGAACGAGAACGCAGACACGATTCGTGCGAAAGTATTG |  |
| *hem*-MR2 | CTCTTCTAAGTTATTGCTGC |  |
| pLP-UR | CAGGAACACTTAACGGCTGAC | Vector-specific internal primer |
| *hem*-TF | ATCGATTGCTATGCTGAATGG | Test insertional mutant with *hem*-TF/pLP-UR and test deletion mutant with *hem*-TF/*hem*-TR. |
| *hem*-TR | ACGAGCCTACTCATACCGTAG |  |
| *degS*-MF1 | TCCAACGTGGAGAGAGAACGC | Get DNA fragment containing two homologous regions of *degS* gene through overlap PCR |
| *degS*-MR1 | ATGATCCCACCGACATGCTCATTGACACCGGATCCTAAACCTTGAATC |  |
| *degS*-MF2 | GATTCAAGGTTTAGGATCCGGTGTCAATGAGCATGTCGGTGGGATCAT |  |
| *degS*-MR2 | AGTTAAACGTCGCGGTGCAGT |  |
| *degs*-TF | GAGTTGCGTGCGATTATGGA | Test insertional mutant with *degS*-TF/pLP-UR and test deletion mutant with *degS*-TF/*degS*-TR. |
| *degs*-TR | GTGACCGTGAGCGTCTACAGG |  |
| *vasC*-MF1 | CAGCATACGTGGACCTCGTCAG | Get DNA fragment containing two homologous regions of *vasC* gene through overlap PCR |
| *vasC*-MR1 | CTTCTGACGTGGCGAACTGCATATCATCCAGTAAGGAGGTCGAACTG |  |
| *vasC*-MF2 | CAGTTCGACCTCCTTACTGGATGATATGCAGTTCGCCACGTCAGAAG |  |
| *vasC*-MR2 | TCGTAGTCGGACGACATAAACATCG |  |
| *vasC*-TF | CAGATGGCGTATGACCTCGA | Test insertional mutant with *vasC*-TF/pLP-UR and test deletion mutant with *vasC*-TF/*vasC*-TR. |
| *vasC*-TR | CTGGAGTCAGTACATAGTCGTAG |  |
| *ascS*-MF1 | CACATTCGTGAAACCGAAGCGAT | Get DNA fragment containing two homologous regions of *ascS* gene through overlap PCR |
| *ascS*-MR1 | TGGTGTAAATCGTCGTAACTCATCGTGCAGGATTCATTCTCTCTCCTTC |  |
| *ascS*-MF2 | GAAGGAGAGAGAATGAATCCTGCACGATGAGTTACGACGATTTACACCA |  |
| *ascS*-MR2 | TTGGGTTGAACATGCTTGCCAT |  |
| *ascS*-TF | GCCAGCGTTGAGGGCCTGAT | Test insertional mutant with *ascS*-TF/pLP-UR and test deletion mutant with *ascS*-TF/*ascS* -TR. |
| *ascS*-TR | TCAATAACACACTCGCGATGG |  |
| *pilO*-MF1 | TCGCGGAGCTTGAACACAG | Get DNA fragment containing two homologous regions of *pilO* gene through overlap PCR |
| *pilO*-MR1 | CGTCTTTAAAGGTGATGATGCGTGGCACTACAAGCTGAGGCAGTAATGG |  |
| *pilO-*MF2 | CCATTACTGCCTCAGCTTGTAGTGCCACGCATCATCACCTTTAAAGACG |  |
| *pilO*-MR2 | CTAACTTGCCAGATCTCGCTCG |  |
| *pilO-*TF | GAAGAGCATCGTCGTCGATT | Test insertional mutant with *pilO*-TF/pLP-UR and test deletion mutant with *pilO*-TF/*pilO*-TR. |
| *pilO*-TR | TCACTTTGTAAACCGTGCCAT |  |
| *impB*-MF1 | GGCGAGTGACAAAATGATCAAGG | Get DNA fragment containing two homologous regions of *impB* gene through overlap PCR |
| *impB*-MR1 | CTTGGCCTAACTTGAGTTCATCAGCCTTCAGTGATGTGCCTTCTGTTT |  |
| *impB*-MF2 | AAACAGAAGGCACATCACTGAAGGCTGATGAACTCAAGTTAGGCCAAG |  |
| *impB*-MR2 | TGGCTTTGATCGAACTCCACTG |  |
| *impB*-TF | GATAGTGCGTGTGAAGAAGTC | Test insertional mutant with *impB*-TF/pLP-UR and test deletion mutant with *impB*-TF/*impB*-TR. |
| *impB*-TR | CATATGGCTCACCACCAGCA |  |

*The bases underlined represent restriction sites.
